# COVID-19 in nursing homes: Geographic diffusion and regional risk factors from January 1 to July 26, 2020 of the pandemic

**DOI:** 10.1371/journal.pone.0308339

**Published:** 2024-08-15

**Authors:** Sue C. Grady, Amanda Pavan, Zhang Qiong, Portelli Rachael, Arika Ligmann-Zielinska

**Affiliations:** 1 Department of Geography, Environment, and Spatial Sciences, Michigan State University, East Lansing, Michigan, United States of America; 2 Newborn Screening Section, Minnesota Department of Health, Minneapolis, Minnesota, United States of America; 3 Division of Public Health, Henry Ford Health System, Detroit, Michigan, United States of America; 4 Department of Geography and Geographic Information Science, University of Illinois at Urbana-Champaign, Urbana, Champaign, Illinois, United States of America; United States Environmental Protection Agency, UNITED STATES OF AMERICA

## Abstract

**Background:**

COVID-19 deaths in nursing homes accounted for 30.2% of all COVID-19 deaths in the United States during the early weeks (1-January to 26-July, 2020) of the pandemic. This study presents the geographic diffusion of COVID-19 cases and deaths in nursing homes during this time period, while also providing explanation of regional risk factors.

**Methods and findings:**

Nursing home COVID-19 data on confirmed cases (n = 173,452) and deaths (n = 46,173) were obtained from the Centers for Medicare and Medicaid Services. Weekly COVID-19 case counts were spatially smoothed to identify nursing homes in areas of high COVID-19 infection. Bivariate spatial autocorrelation was used to visualize High vs. Low-case counts and related deaths. Zero-inflated negative binomial models were estimated within Health and Human Service (HHS) Regions at three-week intervals to evaluate facility and area-level risk factors. The first reported nursing home resident to die of COVID-19 was in the state of Washington on 28-February, 2020. By 24-May, 2020 there were simultaneous epicenters in the Northeast (HHS Regions 1 and 2) and Midwest (HHS Region 5) with diffusion into the South (HHS Regions 4 and 6) from 15-June to 5-July, 2020. The case-fatality rate was highest from 25-May to 14-June, 2020 (30.9 deaths per 1000 residents); thereafter declining to 24.1 (15-June to 5-July, 2020) and 19.4 (6-July to 26-July, 2020) (overall case-fatality rate 1-January to 26-July = 26.6). Statistically significant risk factors for COVID-19 deaths were admission of patients with COVID-19 into nursing homes, staff confirmed infections and nursing shortages. COVID-19 deaths were likely to occur in nursing homes in high minority and non-English speaking neighborhoods and neighborhoods with a high proportion of households with disabilities.

**Conclusions:**

Enhanced communication between HHS regional administrators about “lessons learned” could provide receiving state health departments with timely information to inform clinical practice to prevent premature death in nursing homes in future pandemics.

## Introduction

The first case of COVID-19 isolated in a nursing home occurred on 28-February, 2020. The World Health Organization (WHO) declared COVID-19 a global pandemic on 11-March, 2020. On 13-March, 2020, the Centers for Medicare & Medicaid Services (CMS) restricted visitation of all visitors and non-essential health care personnel from entering nursing homes under the “Guidance for Limiting the Transmission of COVID-19 for Nursing Homes” [1, 2]. Protocols for the use of personal protective equipment (PPE) for health care workers and surveyors and facility guidelines were established including: SARS-CoV-2 testing guidance; when nursing homes should consider transferring a resident with suspected or confirmed COVID-19 infection to a hospital; and when a nursing home should accept a resident diagnosed with COVID-19 from a hospital [3]. National surveillance from mandatory reporting to the CMS of COVID-19 suspected and confirmed cases and deaths showed that from 1-January to 26-July, 2020 there were 173,452 confirmed COVID-19 cases reported from nursing homes, including 46,173 COVID-19 related deaths (case-fatality rate = 26.6 per 100 nursing home residents). By 26-July, 2020 nursing home deaths accounted for 30.2% of total COVID-19 deaths in the United States [1, 4].

The earlier studies on the impact of the pandemic on nursing home residents used pre-surveillance sources of COVID-19 case and death data and the CMS-COVID-19 Nursing Home Data for the United States [5–10], state-specific case studies for Connecticut [11], California [12, 13], a 23-state study using data from health departments on 8,943 nursing homes [14], county and facility-level studies [15] and a facility-specific case study in Washington [16]. Across these studies, there were several common facility-level risk factors for COVID-19 transmission, included larger facility size [5, 8, 12], nursing staff shortages [7–9, 11, 12, 14], staff who worked while symptomatic [16], or staff who worked in more than one facility [16], limited testing availability [16], for-profit vs. non-profit ownership [5, 9, 10, 13, 14], a higher percentage of Medicaid residents [5, 11, 14] and a higher number of total facility deficiencies or penalties [8, 12, 14]. In terms of patient demographics, Li et al. [11] found that nursing homes in Connecticut with a high proportion of minority residents had 15.0% higher confirmed COVID-19 cases than comparable facilities. He et al. [13] also found that nursing homes in California with a higher proportion of non-white residents had a higher odds of COVID-19 cases and deaths. None of these studies specifically investigated the transfer of hospitalized patients with COVID-19 to nursing homes for additional care, and the potential of amplifying spread in nursing homes.

The social context of nursing homes was also an important risk factor for COVID-19 transmission. Two studies, Abrams et al. [5] and Travers et al. [15] found that nursing homes in areas with high vs. low shares of Black residents had a higher odds of COVID-19 presence; however, White et al. [6] did not find significant racial differences in county-level COVID-19 risk. Abrams et al. [5] found that nursing homes in urban vs. rural areas had a higher odds of COVID-19 presence and as COVID-19 increased in the community, the likelihood of a nursing home outbreak also increased. Chatterjee et al. [14] and Hege et al. [9] also found an increase in COVID-19 reporting in nursing homes in counties with higher COVID-19 rates. Traverse et al. [15] found county-level resources, rurality and counties with a high proportion of Black residents in part explained high rates of COVID-19 and deaths in nursing homes nationwide.

These studies demonstrated the need to understand facility- and contextual-level risk factors for COVID-19 within and across nursing homes, to thereby, reduce the likelihood of premature deaths in future pandemics.

### Purpose of study

The purposes of this study were to utilize the COVID-19 nursing home surveillance data to (a) document the spatial patterns and diffusion of confirmed COVID-19 cases and deaths from 1-January to 26-July, 2020 and (b) to examine facility and contextual-level risk factors for COVID-19 deaths in nursing to explain these trends. To ensure an adequate sample (and power) to detect risk factors for COVID-19 deaths, this study investigated nursing home risks within the U.S. Health and Human Service (HHS) regions [17] at three-week intervals. The findings from this retrospective cross-sectional study were intended to inform HHS regional administrators and state health departments to prevent transmission and premature deaths in U.S. nursing homes in future pandemics.

## Materials and methods

### Data

Nursing home COVID-19 data on confirmed cases (n = 173,452) and deaths (n = 46,173) between 1-January to 26-July, 2020 were obtained from the Nursing Home COVID-19 Public File Centers for Medicare and Medicaid Services (CMS)—Division of Nursing Homes/Quality, Safety, and Oversight Group/Center for Clinical Standards and Quality (Data.CMS.gov) [4]. These data included reports by nursing homes to the CDC’s National Healthcare Safety Network (NHSN) System COVID-19 Long Term Care Facility Module [18]. Individual records were from each certified Medicare skilled nursing facility/Medicaid nursing and long-term care facility with data for each collection week. The authors did not have information that could identify individuals within nursing homes and thus the data used for this study were exempt from IRB review.

The dependent variable in subsequent statistical analyses was the count of COVID-19 deaths among residents (ResidentsWeeklyCOVID19Deaths) in nursing homes within HHS regions. Explanatory variables of interest included (a) the count of COVID-19 confirmed and suspected cases, staff diagnosed with COVID-19 and admissions of COVID-19 patients from hospitals into nursing homes to investigate levels of infectivity in nursing homes and the potential of COVID-19 transmission, (b) the count of nursing shortages to assess a potential barrier to care, and (c) the facility conditions, such as the availability of testing and the presence or absence of a ventilator dependent unit in the nursing home.

**Fig 1:**
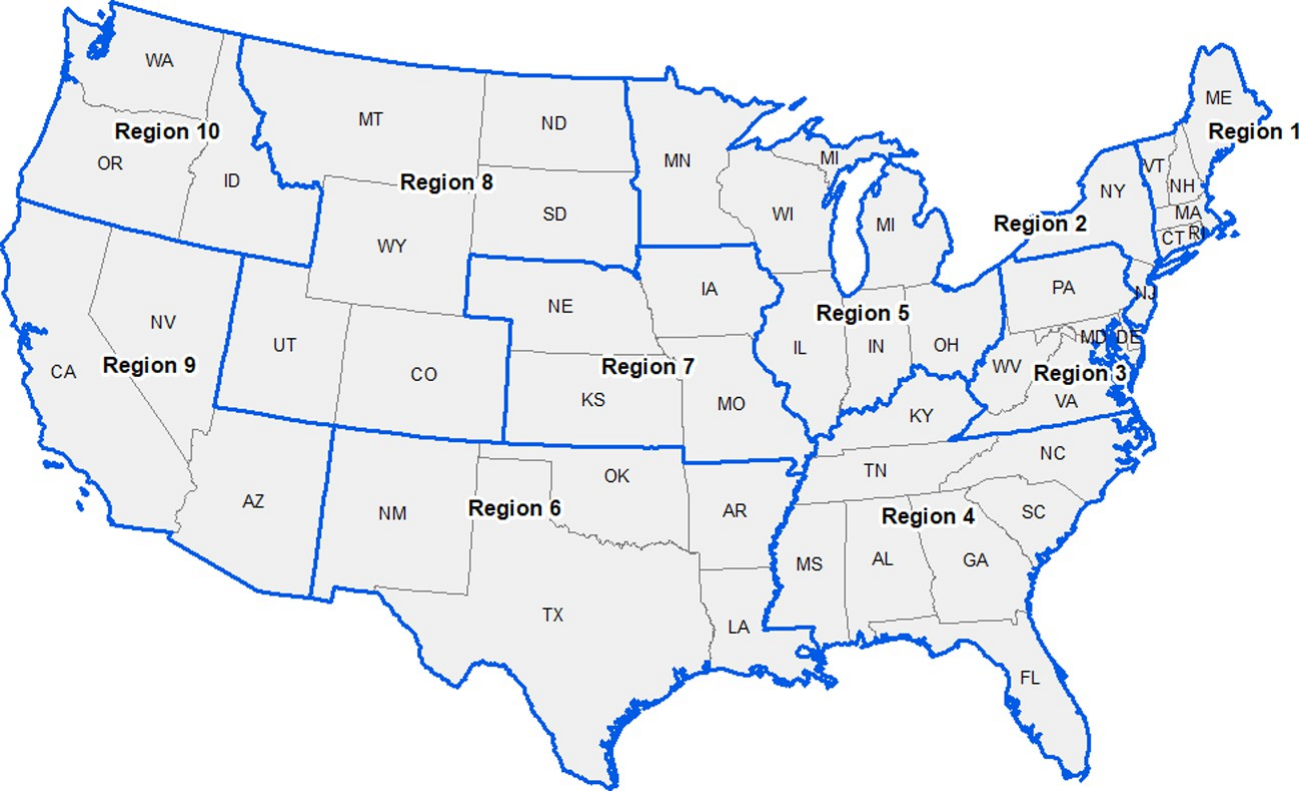
Reference map of states within U.S. Department of Health and Human Services (HHS) Regions. Source: [17].

Also, the neighborhood location of nursing homes was used to assess potential vulnerability following studies that have shown the importance of understanding contextual-level risks and the potential of community level COVID-19 infectivity rates (1 = urbanized-area, 0 = all others; 1 = urban-cluster, 0 = all others; 1 = rural = 1, 0 = all others) with urbanized areas defined as 50,000 or more people; urban clusters defined as at least 2,500 and less than 50,000 people; and rural areas having less than 2,500 people. Social vulnerability was measured using the Center for Disease Control’s Social Vulnerability Index [19, 20] which included four continuous themes (1) low socio-economic status (including low education, employment and poverty), (2) household composition (including a high proportion of dependency, single parenting and disability), (3) high minority and non-English speaking residents, and (4) crowded housing with low access to automobile transportation. For each census tract, the percentile rank across all tracts for each of the four themes was used in this study. The nursing home point locations were spatially joined to the 2010 urban boundary [21] and the Social Vulnerability Index at the census tract level in ArcGIS v. 10.8 [22] to assign these contextual attributes to each nursing home. Finally, new variables were created including the bed-level of the nursing home using the number of beds listed, and if missing, the number of occupied beds was used (base = <50 beds, small = 50–99 beds, mid-sized = 100–249 beds, and large = 250+ beds [23]. Veteran’s Nursing Homes were also coded (1 = yes, 0 = no). Nursing homes within and across states were studied within HHS Regions (see Fig 1).

### Spatial diffusion

The nursing home’s geocoded point locations were used to spatially smooth the confirmed case counts using kernel density in ArcGIS v. 10.8 [22] with a default grid cell size and 1 square-mile bandwidth parameters. Bivariate spatial autocorrelation was used to detect nursing homes with clusters of High-case counts + High-deaths, Low-case counts + High-deaths and Low-case counts + Low-deaths. These case-death clusters were overlayed onto the spatially smoothed case data to visual the spatial patterns of COVID-19 diffusion across nursing homes. These spatial analyses were conducted for the time period 1-January to 24-May, 2020 and weekly thereafter through 26-July, 2020.

### Statistical analysis

An examination of the weekly counts of COVID-19 deaths revealed that they were highly right skewed with a high percentage of records listed as zeros. Zeros in the data set were thus viewed as structural -i.e., the zeros were due to a sub-population of residents who were not at risk of COVID-19 transmission; or sample of residents who were at risk of COVID-19 but still produced a zero outcome perhaps due to testing variability [24]–e.g., antibody testing too soon post-exposure. In this study, zeros were modeled as a latent variable as outlined below.

Zero-inflated negative binomial (ZINB) models were estimated using ‘proc countreg’ in SAS v. 9.3. [25] to account for overdispersion (variance substantially larger than mean) and excess of zeros [26]. If significant dispersion was not observed, a zero-inflated Poisson (ZIP) model was estimated. These models had two components by which to estimate each of the two distributions: negative binomial model to predict non-zero or counts of COVID-19 deaths and a logistic model to predict the excessive zeros. The count of occupied beds in nursing homes was used as an offset in the model. Bivariate Pearson correlation for the continuous counts of data (proc corr) was used to assess whether a statistically significant linear relationship existed between two continuous variables and the direction and strength of the relationship. These findings informed the parameterization of the ZINB and ZIP models; in particular, those coefficients with large standard errors. The maximum log-likelihood was used to fit the model. The smallest Akaike Information Criterion (AIC) and Schwartz’s Bayesian information criterion (SBC) were used to identify the best fit model.

These data were grouped into four distinct time periods for statistical analysis at the HHS regional level 1-January to 24-May, 2020 and thereafter, 3 week intervals 25-May to 14-June, 2020, 15-June to 5-July, 2020 and 6-July to 26-July, 2020. These groupings (excluding the first time period that included back-logged records) were derived from knowledge of the disease continuum -i.e., 2–14 days from exposure to symptoms, plus one week to potential death. The benefits of using a 21-day window rather than a weekly time-series were higher case counts and power for statistical analyses, and it accounted for potential variation in reporting lags.

## Results

**Fig 2:**
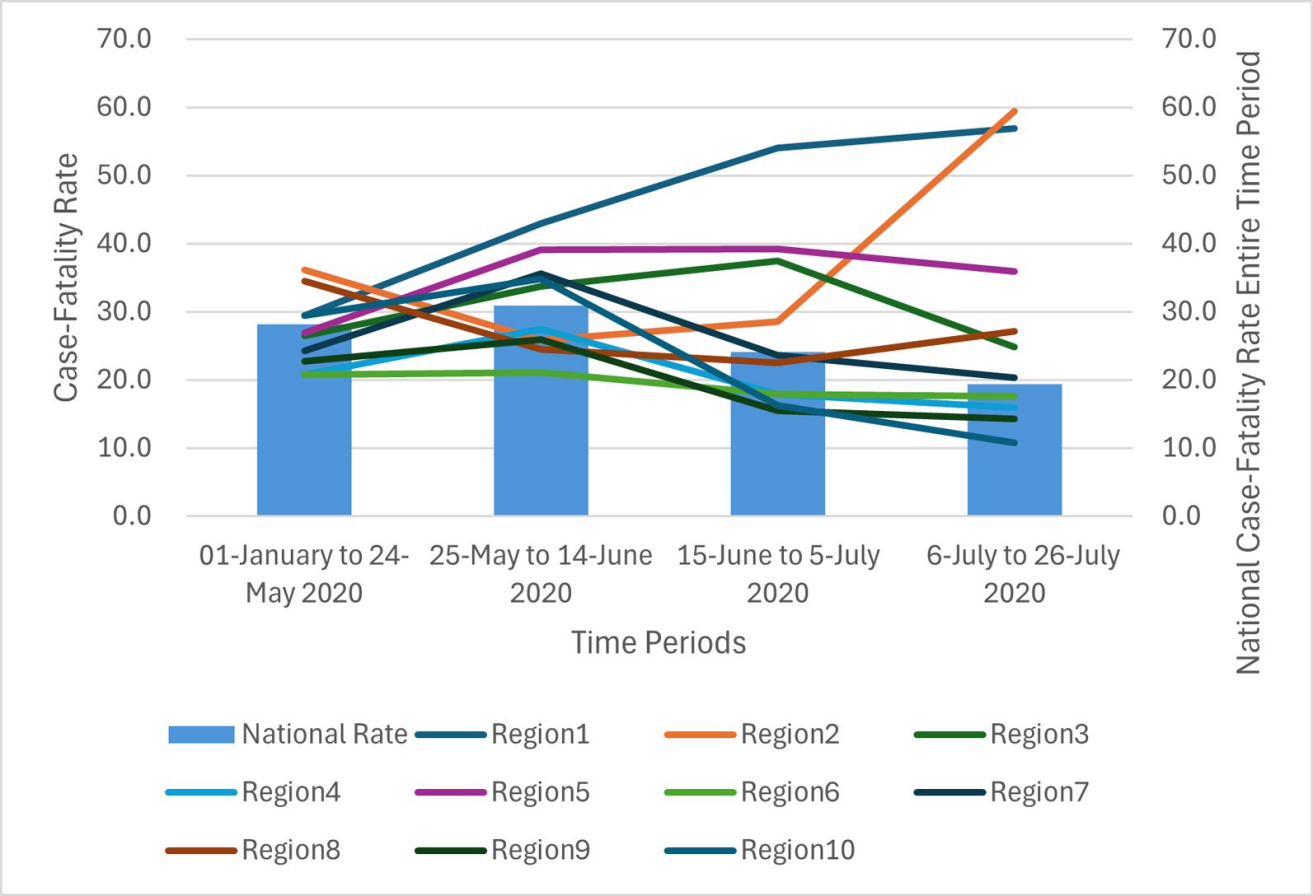
COVID-19 case-fatality rates by U.S. Health and Human Services (HHS) Regions, 1-January to 26-July, 2020.

The national case-fatality rate (COVID-19 deaths per 100, confirmed COVID-19 cases) from 1-January to 26-July, 2020 was 26.6 with rates in Regions 1, 2, 3, 5 and 8 higher than the national average. Fig 2 (*if accepted, production will need this reference to link the reader to the figure) shows the summary data on nursing home case-fatality rates by HHS Region for four time periods. Those HHS Regions that were higher than the national averages for 1-January to 24-May, 2020 (28.2) were Regions 1, 2, 8 and 10, 25-May to 14-June, 2020 (30.8) were Regions 1, 3, 5, 7 and 10, June 15 to 5-July, 2020 (24.1) were Regions 1, 2, 3, and 5 and 6-July to 26-July, 2020 (19.4) were Regions 1, 2, 3, 5 and 7. These findings demonstrate that nursing home case-fatality rates fluctuated with epicenters in the Northeast and Midwest early in the pandemic. The case-fatality rates for states within these HHS Regions are presented in S1 Table.

### 1-January to 24-March, 2020

**Fig 3:**
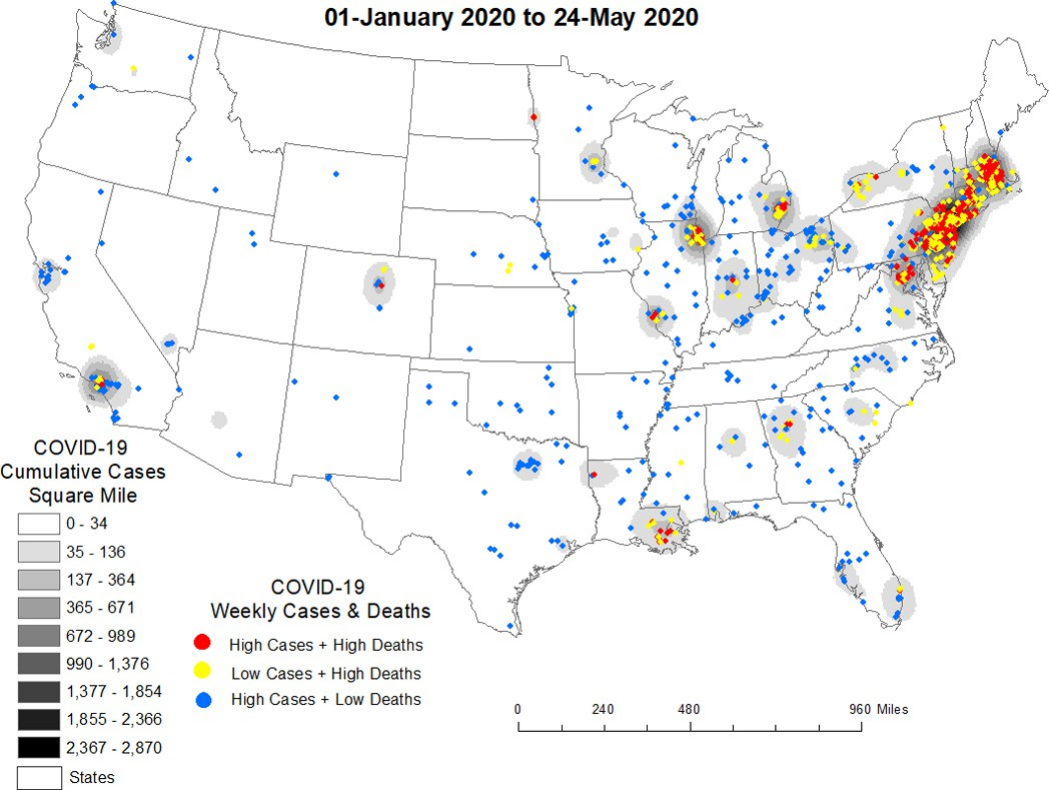
Nursing home COVID-19 diffusion from 1-January to 24-May, 2020.

Between 1-January to 24-May, 2020 nursing homes with High-case counts + High-deaths and Low-case counts + High-deaths were clustered in states along the eastern seaboard, in urban areas of the Midwest and dispersed in some cities in the South. Clusters of nursing homes with High-case counts + Low-deaths were largely outside of cities and in rural areas (see Fig 3).

Table 1 shows factors that underly these trends from 1-January to 24-May, 2020. Specifically, confirmed COVID-19 infection among residents and staff diagnosed with COVID-19 were significantly associated with COVID-19 deaths in nursing homes in HHS Regions 1, 2, 3, 5 and 9. Admission of COVID-19 patients from hospitals into nursing homes was also a significant risk factor in HHS Regions 2 and 5. There were significantly higher deaths in mid-sized nursing homes (100–249 beds) compared to small nursing homes (<50 beds) in HHS Regions 2 and 3—a phenomena not seen in large nursing homes (250+ beds) except in Region 5. Neighborhood social vulnerability was not a significant risk factor for COVID-19 deaths in nursing homes from 1-January to 24-May, 2020 of the pandemic.

**Table 1:** Estimating nursing home COVID-19 deaths by U.S. Health and Human Service (HHS) Regions, 1-January to 24-May, 2020: Zero-inflated negative binomial models^1^.

Health & Human Service Regions	Region 1			Region 2			Region 3			Region 5			Region 9		
	B	SE	p-value	B	SE	p-value	B	SE	p-value	B	SE	p-value	B	SE	p-value
Intercept	1.1788	0.1997	<0.0001	0.3282	0.3026	0.2779	0.2353	0.2656	0.3756	0.3670	0.2445	0.1333	-0.5668	-0.5340	0.2885
AdmissionsCovid19	0.0062	0.0029	0.0323	0.0118	0.0027	<0.0001	0.0043	0.0047	0.3510	0.0109	0.0035	0.0015	0.0194	0.0076	0.0106
SuspectedCovid19	0.0011	0.0018	0.5077	0.0060	0.0020	0.0021	0.0027	0.0021	0.2003	0.0062	0.0030	0.0410	-0.0087	0.0037	0.0188
ConfirmedCovid19	0.0136	0.0017	< 0.0001	0.0138	0.0016	<0.0001	0.0191	0.0019	< 0.0001	0.0227	0.0024	<0.0001	0.0157	0.0045	0.0004
Shortage Nursing Staff	-0.6809	0.0945	0.4713	-0.1949	0.1079	0.0708	-0.0562	0.1023	0.5823	-0.1902	0.0898	0.0341	0.4995	0.3173	0.1155
StaffConfirmedCovid19	0.017	0.0035	<0.0001	0.0170	0.0029	<0.0001	0.0108	0.0040	0.0072	0.0164	0.0038	<0.0001	0.0302	0.0073	<0.0001
Beds 50–100 vs. < 50	0.1746	0.1762	0.3216	0.7700	0.3017	0.0107	0.702	0.2406	0.0035	0.3137	0.2075	0.1306	0.2716	0.2370	0.2499
Beds 100–249 vs. < 50	0.1512	0.1632	0.3541	0.7468	0.2758	0.0068	0.6897	0.2298	0.0027	0.4424	0.2024	0.0288	0.1003	0.2353	0.6700
Beds 250+ vs. < 50	-	-	-	0.4043	0.2915	0.1655	0.3598	0.2774	0.1945	0.5123	0.2675	0.0555	-	-	-
Veterans Homes	-	-	-	0.2798	0.3735	0.4537	0.0622	0.4983	0.9007	-	-	-	-	-	-
*SV-Socioeconomic	-0.5075	0.2294	0.0270	-0.0084	0.2207	0.9696	-0.4904	0.2196	0.0255	0.3225	0.2130	0.1300	-0.2662	0.2353	0.6700
SV-Household characteristics	-0.1284	0.1742	0.4608	-0.0788	0.1818	0.6646	0.3012	0.1883	0.1096	-0.1729	0.1818	0.3416	-0.3612	0.3349	0.2807
SV-Race & Ethnicity	0.3458	0.2196	0.1153	0.0441	0.2137	0.8365	0.1174	0.1787	0.5318	-0.1383	0.1852	0.4552	-0.2925	0.4867	0.5479
SV-Housing/transportation	0.0182	0.1838	0.9209	0.1717	0.1926	0.3725	0.2412	0.1937	0.2131	-0.0946	0.1791	0.5976	0.8077	0.4229	0.0562
Urban Area	-	-	-	-	-	-	-	-	-	-	-	-	0.8133	0.3859	0.0351
Inf_Intercept	2.6423	0.2329	<0.0001	2.0182	0.2182	<0.0001	3.3539	0.2388	<0.0001	3.4329	0.1615	<0.0001	3.7883	0.2954	<0.0001
Inf_AdmissionsCovid19	-0.8321	0.1662	<0.0001	-0.5635	0.1497	0.0002	-0.6904	0.2467	0.0051	-0.3936	0.0887	<0.0001	-1.0664	0.2419	<0.0001
Inf_SuspectedCovid19	-0.0317	0.0250	0.2048	-0.0501	0.0383	0.1895	0.0349	0.0307	0.2551	-0.0410	0.0276	0.1368	-0.0478	0.0217	0.0274
Inf_ConfirmedCovid19	-0.8476	0.2162	<0.0001	-0.5571	0.1478	0.0002	-1.1759	0.3253	0.0003	-1.9108	0.4046	<0.0001	-1.4998	0.3194	<0.0001
Alpha	0.5141	0.0488	<0.0001	0.7602	0.0612	<0.0001	0.4892	0.0568	<0.0001	0.8342	0.0718	<0.0001	1.0002	0.1366	<0.0001
Observed Mean	48.52			41.83			70.30			79.66			83.58		
Modelled Mean	47.47			40.35			69.18			78.98			82.41		
AIC	3115			3866			2755			4502			1700		
SBC	3195			3957			2853			4616			1793		

^1^Intercept Negative Binomial Model to estimate COVID-19 death counts offset by facility’s occupancy; Inf_Intercept logistic models to estimate zeros (SAS v.9.3) controlling for testing and presence vs. absence of a ventilatory unit.

^2^SV = Social Vulnerability.

### 25-May to 14-June, 2020

**Fig 4:**
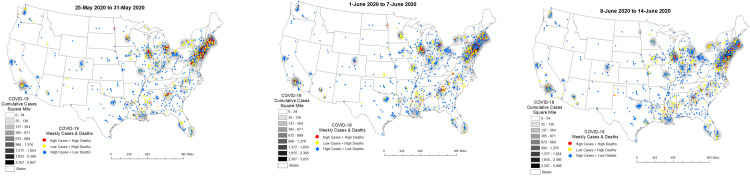
**a–c**. Nursing Home COVID-19 Diffusion from 25-May to 14-June, 2020.

From 25-May to 31-May, 2020 clusters of High-case counts + High-deaths and Low-case counts + High-deaths expanded in states along the eastern seaboard, in urban areas in the Midwest and more cities in the South. There were however, an increase in clusters of High-case counts + Low-deaths also emerging in the Northeast (see Fig 4A). These patterns persisted the following week (1-June to 7-June, 2020) and the week thereafter (8-June to 14-June, 2020) (see Fig 4B and 4C) where greater expansive diffusion of COVID-10 cumulative cases per square mile within states was also observed in the Northeast and Midwest.

Table 2 shows factors that underly these trends from 25-May to 14-June, 2020. While confirmed COVID-19 cases was a significant risk factor for COVID-19 deaths in HHS Regions 2, 4 and 5 staff confirmed with COVID-19 was a significant risk factor in HHS Regions 1, 2, 3, 4 and 5 demonstrating diffusion of staff confirmed infections into the South. The admission of COVID-19 patients from hospitals to nursing homes continued to be a risk factor in Region 5. During this time-period, nursing shortages also emerged as a significant risk factor in Region 5. In HHS Region 2 the risk of death in mid- (100–249 beds) and large- (>250+ beds) sized nursing homes was not significantly greater than in small (<50 beds) nursing homes which was a change from the prior reporting period. During this time-period neighborhood social vulnerability emerged as a risk factor with nursing homes in Region 3 experiencing higher deaths in neighborhoods with high dependency, single parent households and households with disabilities. In Region 4 COVID-19 deaths were significantly high in nursing homes in high minority and low-English speaking neighborhoods.

**Table 2:** Estimating nursing home COVID-19 deaths by U.S. Health and Human Service (HHS) Regions, 25-May to 14-June 2020: Zero-inflated negative binomial models^1^.

Health & Human Services	Region 1			Region 2			Region 3			Region 4			Region 5		
	B	SE	p-value	B	SE	p-value	B	SE	p-value	B	SE	p-value	B	SE	p-value
Intercept	-2.2469	0.3968	<0.0001	-0.4027	0.3461	0.2445	-1.2453	0.2879	<0.0001	-2.2910	0.3681	<0.0001	-1.4640	0.2679	<0.0001
AdmissionsCovid19	0.0214	0.0217	0.3341	0.0400	0.0166	0.0156	0.0479	0.0211	0.0228	0.0386	0.0157	0.0142	0.0419	0.0119	0.0004
SuspectedCovid19	0.0636	0.0183	0.0005	-0.0015	0.0175	0.9326	0.0079	0.0144	0.5851	0.0040	0.0132	0.7643	0.0250	0.0122	0.0409
ConfirmedCovid19	0.0073	0.0091	0.4250	0.0397	0.0077	<0.0001	0.0191	0.1328	0.2637	0.0517	0.0095	<0.0001	0.0396	0.0085	<0.0001
Shortage Nursing Staff	0.2201	0.1225	0.0724	0.2552	0.1264	0.0435	0.1484	0.1328	0.2637	0.1516	0.1258	0.2283	0.3977	0.0999	<0.0001
StaffConfirmedCovid19	0.1222	0.0247	<0.0001	0.0284	0.0094	0.0024	0.0514	0.0139	0.0002	0.0444	0.0150	0.0030	0.0728	0.0148	<0.0001
Beds 50–100 vs. < 50	1.3570	0.3643	0.0002	-0.2559	0.3553	0.4714	0.4617	9.2712	0.0887	0.7057	0.3141	0.0247	0.7220	0.2314	0.0018
Beds 100–249 vs. < 50	1.8619	0.3562	<0.0001	0.5911	0.3106	0.0571	0.8357	0.2517	0.0009	0.9053	0.2974	0.0023	1.0899	0.2306	<0.0001
Beds 250+ vs. < 50	2.1080	0.4369	<0.0001	0.6330	0.3277	0.0534	0.8630	0.3051	0.0047	-	-	-	1.0493	0.2907	0.0003
Veterans Homes	-	-	-	0.2806	0.5090	0.5815	0.8354	0.5364	0.1993	-	-	-	-	-	-
*SV-Socioeconomic	-0.0003	0.3134	0.9992	-0.0718	0.2626	0.7846	-0.4318	0.3096	0.1632	-0.1508	0.3130	0.6299	-0.2044	0.2376	0.3898
SV-Household characteristics	-0.4007	0.2399	0.0950	0.0591	0.2261	0.7939	1.2052	0.2676	<0.0001	-0.3341	0.2453	0.1738	-0.2021	0.2080	0.3311
SV-Race & Ethnicity	0.5898	0.2962	0.0468	-0.5619	0.2577	0.0292	-0.1729	0.2486	0.4686	1.1885	0.2543	<0.0001	0.4952	0.2095	0.0181
SV-Housing/transportation	-0.6028	0.2350	0.0103	-0.9246	0.2227	<0.0001	-0.1777	0.2670	0.5058	0.5293	0.2782	0.0571	-0.2706	0.1931	0.1610
Urban Area	0.3751	0.1732	0.0304	-	-	-	-	-	-	-	-	-	-	-	-
Urban Cluster	-	-	-	-	-	-	-0.3647	0.1712	0.0332	-	-	-	0.3289	0.1379	0.0170
Rural	-	-	-	-	-	-	-	-	-	0.3348	0.1394	0.0163	-	-	-
Inf_Intercept	1.0278	0.1485	<0.0001	1.0791	0.1654	<0.0001	2.2232	0.1245	<0.0001	2.8278	0.1235	<0.0001	2.3267	0.1013	<0.0001
Inf_AdmissionsCovid19	-0.7223	0.1715	<0.0001	-0.9011	0.2715	0.0009	-1.5394	0.2301	<0.0001	-2.4824	0.2813	<0.0001	-1.9360	0.2650	<0.0001
Inf_SuspectedCovid19	0.2465	0.0966	0.0107	-0.6170	0.2745	0.0246	0.0621	0.0485	0.2003	-0.2296	0.0694	0.0009	-0.0366	0.0629	0.5605
Inf_ConfirmedCovid19	-2.4846	0.5885	<0.0001	-1.0968	0.3052	0.0003	-2.7577	0.4122	<0.0001	-2.2606	0.3593	<0.0001	-3.0470	0.3652	<0.0001
Alpha	1.0295	0.1405	<0.0001	1.7027	0.1925	<0.0001	1.4933	0.1573	<0.0001	2.0430	0.2051	<0.0001	2.0126	0.1673	<0.0001
Observed Mean	81.45			80.73			87.80			93.51			92.00		
Modelled Mean	82.23			81.12			88.23			93.62			92.09		
AIC	3224			3803			3612			4090			6237		
SBC	3335			3916			3737			4216			6373		

^1^Intercept Negative Binomial Model to estimate COVID-19 death counts offset by facility’s occupancy; Inf_Intercept logistic models to estimate zeros (SAS v.9.3) controlling for testing and presence vs. absence of a ventilatory unit. ^2^SV = Social Vulnerability.

### 15-June to 5-July 2020

**Fig 5:**
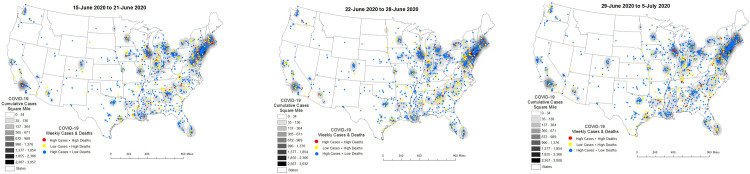
**a–c**. Nursing Home COVID-19 Diffusion from 15-June to 5-July, 2020.

From 15-June to 21-June, 2020 clusters of High-case counts + High-deaths and Low-case counts + High-deaths in nursing homes began to diminish along the eastern seaboard with clusters of High-case counts + Low-deaths more pronounced. As the COVID-19 cases per square mile diffused across states in the Northeast and Midwest however, there was also an increase in Low-case counts + High-deaths in areas outside of the original epicenters. A similar phenomenon was observed in the South (see Fig 5A). These north-south trends persisted through the next week (22-June to 28-June, 2020) and the week thereafter (29-June to 5-July, 2020) (see Fig 5B and 5C).

Table 3 shows factors that underly these trends from 15-June to 5-July, 2020. Confirmed COVID-19 cases persisted as a significant risk factor for COVID-19 deaths in HHS Regions 2, 4 and 5 with staff confirmed COVID-19 a significant risk factor in HHS Regions 3, 5 and 6. Furthermore, the admission of COVID-19 patients from hospitals to nursing homes emerged in HHS Regions 4 and 6—these findings demonstrating the diffusion of known risk factors for COVID-19 deaths in nursing homes into the South. Nursing shortages also emerged as a significant risk factor for COVID-19 deaths in HHS Region 4 while persisting in HHS Region 5. In HHS Regions 2 and 6 the risk of death in mid- (100–249 beds) and large- (> 250+ beds) sized nursing homes was not significantly greater than in small (< 50 beds) nursing homes—this phenomenon persisting in HHS Region 2 but new in HHS Region 6. Finally, risk factors relating to social vulnerability persisted in Region 3 with higher COVID-19 deaths continuing in neighborhoods with high dependency, single parent households and households with disabilities. In Region 5 COVID-19 deaths were significantly elevated in high minority and low-English speaking neighborhoods. In Region 4 COVID-19 deaths in nursing homes persisted in neighborhoods with high minority and low-English speaking populations. In Region 6 COVID-19 deaths in nursing homes were significantly high in urban areas.

**Table 3:** Estimating nursing home COVID-19 deaths by U.S. Health and Human Service (HHS) Regions, 15-June to 5-July 2020: Zero-inflated negative binomial models^1^.

Health & Human Services	Region 2			Region 3			Region 4			Region 5			Region 6		
	B	SE	p-value	B	SE	p-value	B	SE	p-value	B	SE	p-value	B	SE	p-value
Intercept	-2.1022	0.6231	0.0007	-2.4048	0.5408	<0.0001	-3.2875	0.6619	<0.0001	-3.7657	0.4489	<0.0001	-3.7507	0.6785	<0.0001
AdmissionsCovid19	0.0139	0.0304	0.6466	0.0341	0.0359	0.3421	0.0686	0.0159	<0.0001	-0.0138	0.0352	0.6956	0.1443	0.0472	0.0022
SuspectedCovid19	-0.0281	0.0480	0.5581	-0.0651	0.0300	0.0300	0.0464	0.0154	0.0026	-0.0522	0.0332	0.1166	0.0713	0.0348	0.0404
ConfirmedCovid19	0.0864	0.0264	0.0011	0.0256	0.0192	0.1831	0.0325	0.0100	0.0012	0.0794	0.0265	0.0008	0.0241	0.0140	0.0849
Shortage Nursing Staff	0.3898	0.2000	0.0513	0.2506	0.2171	0.2484	0.4216	0.1261	0.0008	0.4964	0.1530	0.0022	0.4609	0.1661	0.0143
StaffConfirmedCovid19	-0.0157	0.0260	0.5469	0.1223	0.0337	0.0003	0.0198	0.0183	0.2786	0.1378	0.0289	0.0004	0.0871	0.0217	<0.0001
Beds 50–100 vs. < 50	-0.1118	0.6331	0.8598	1.7818	0.5082	0.0005	1.5944	0.6421	0.0130	1.4915	0.3965	0.0002	0.7574	0.5743	0.1872
Beds 100–249 vs. < 50	1.1965	0.5471	0.0287	1.8585	0.4916	0.0002	1.9850	0.6362	0.0018	1.9825	0.3939	<0.0001	1.0559	0.5568	0.0579
Beds 250+ vs. < 50	1.1521	0.5707	0.0435	2.1012	0.5460	0.0001	1.6548	0.7269	0.0228	1.8249	0.4935	0.0002	0.6287	0.9651	0.5148
Veterans Homes	1.1128	0.7248	0.1247	0.0237	0.7241	0.9739	-	-	-	-	-	-	-	-	-
*SV-Socioeconomic	-0.0634	0.4358	0.8843	-0.4660	0.4425	0.2922	0.0526	0.3153	0.8675	0.2527	0.3451	0.4640	0.9172	0.4118	0.0259
SV-Household characteristics	0.5701	0.3580	0.1113	0.7384	0.3916	0.0594	-0.2445	0.2695	0.3643	0.5394	0.3274	0.9885	-0.2241	0.3394	0.5091
SV-Race & Ethnicity	-1.2473	0.4120	0.0025	0.3803	0.3758	0.3116	0.8441	0.2549	0.0009	1.1145	0.3211	0.0005	-0.2607	0.3660	0.2193
SV-Housing/transportation	-0.8449	0.3369	0.0121	-0.8305	0.3734	0.0262	-1.0033	0.2687	0.7089	-0.4259	0.2987	0.1539	0.4706	0.3830	0.2193
Urban Area	0.5625	0.2369	0.0176	-	-	-	-	-	-	0.3552	0.1726	0.0396	1.1138	0.2190	<0.0001
Urban Cluster	-	-	-	-	-	-	0.3199	0.1400	0.0223	-	-	-	-	-	-
Rural	-	-	-	-	-	-	-	-	-	-	-	-	0.6136	0.2423	0.0113
Inf_Intercept	1.2850	0.2346	<0.0001	2.0047	0.1703	0.0262	2.3974	0.1325	<0.0001	2.0937	0.1441	<0.0001	2.2340	0.1779	<0.0001
Inf_AdmissionsCovid19	-1.7927	0.5230	0.0006	-2.4346	0.4714	<0.0001	-2.0073	0.3678	<0.0001	-0.4172	0.2351	0.0760	-2.1527	0.6080	0.0004
Inf_SuspectedCovid19	-0.5690	0.5238	0.2774	-0.3403	0.1307	0.0092	-0.0704	0.0637	0.2688	-0.4172	0.2351	0.0076	-0.0168	0.0595	0.7777
Inf_ConfirmedCovid19	-2.8943	1.3723	0.0349	-2.0303	0.4657	<0.0001	-1.8366	0.3165	<0.0001	-2.1262	0.4332	<0.0001	-2.2627	0.4240	<0.0001
Alpha	2.8672	0.5006	<0.0001	3.6839	0.4866	<0.0001	2.4676	0.2692	<0.0001	4.6618	0.5078	<0.0001	3.0241	0.4039	<0.0001
Observed Mean	91.94			93.08			94.01			95.70			95.26		
Modelled Mean	91.97			93.31			94.13			95.84			95.43		
AIC	1886			2506			3910			3936			2522		
SBC	2005			2626			4042			4072			2657		

^1^Intercept Negative Binomial Model to estimate COVID-19 death counts offset by facility’s occupancy; Inf_Intercept logistic models to estimate zeros (SAS v.9.3) controlling for testing and presence vs. absence of a ventilatory unit. ^2^SV = Social Vulnerability.

### 6-July to 26-July 2020

**Fig 6:**
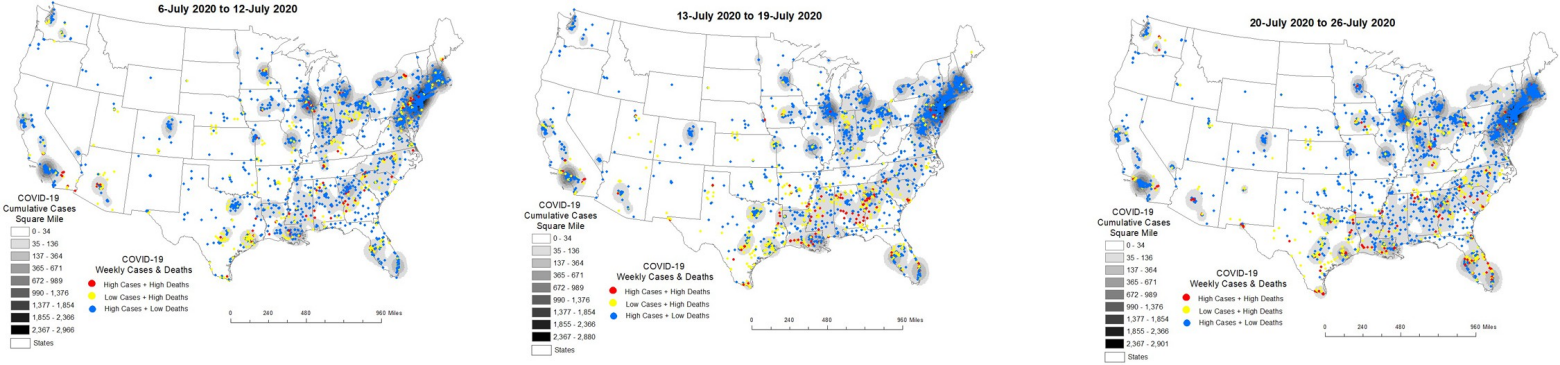
**a–c**. Nursing Home COVID-19 Diffusion from 6-July to 26-July 2020.

From 6-July to 12-July, 2020 clusters of High-case counts + Low-deaths were prominent in the Northeast demonstrating a sustained transition from clusters of High-case counts + High-deaths and Low-case counts + High deaths. Clusters of High- or Low-case counts + High-deaths were largely observed outside of urban areas in the Northeast and Midwest but now dispersed throughout the South and Southwest (see Fig 6A) the following week (13-July to 19-July, 2020) and the week thereafter (20-July to 26-July, 2020) (see Fig 6B and 6C). By 26-July, 2020 the epicenters of nursing home COVID-19 deaths was in the South with emergence into the Southwest.

Table 4 shows factors that underly these trends from 6-July to 26-July, 2020. Confirmed COVID-19 cases and staff confirmed with COVID-19 were significant risk factors for COVID-19 deaths in HHS Regions 4 and 6 of the South. Admissions of COVID-19 patients into nursing homes emerged as a risk factor in HHS Region 3 and continued to be a risk factor in HHS Region 4. Importantly, there was also an increase in COVID-19 deaths in Veteran’s Homes in Region 3. In Regions 5 and 6 large- (> 250+ beds) nursing homes compared to small- (< 50 beds) nursing homes was not a significant risk factor for COVID-19 deaths—this phenomena persisting in HHS Region 6 and reemerging in HHS Region 5. Finally, in the Northeast (Regions 2 and 3) COVID-19 deaths in nursing homes were significantly high in urban areas and neighborhoods with a high dependency, single-parent households and households with disabilities. In HHS Region 3 high COVID-19 deaths were also observed in nursing homes in high minority and low English-speaking residents—a new phenomenon for HHS Region 3.

**Table 4:** Estimating nursing home COVID-19 deaths by U.S. Health and Human Service (HHS) Regions, 6-July to 26-July 2020: Zero-inflated negative binomial models^1^.

Health & Human Services	Region 2			Region 3			Region 4			Region 5			Region 6		
	B	SE	p-value	B	SE	p-value	B	SE	p-value	B	SE	p-value	B	SE	p-value
Intercept	-4.5740	0.8417	<0.0001	-4.0767	0.9041	<0.0001	-1.8235	0.4618	<0.0001	-2.3569	0.3239	<0.0001	-1.6062	0.4343	0.0002
AdmissionsCovid19	-0.1363	0.0859	0.1225	0.1359	0.0507	0.0073	0.0931	0.0145	<0.0001	-0.0257	0.0416	0.5369	0.0321	0.0187	0.0856
SuspectedCovid19	0.5108	0.2247	0.0230	0.0480	0.0311	0.1243	0.0053	0.0092	0.5641	0.1203	0.0323	0.0002	-0.0215	0.0097	0.0263
ConfirmedCovid19	0.0077	0.0523	0.8825	0.0256	0.0186	0.1692	0.0260	0.0058	<0.0001	0.0127	0.0247	0.6057	0.0234	0.0063	0.0002
Shortage Nursing Staff	-0.0870	0.4728	0.8540	-0.2885	0.3200	0.3673	0.5253	0.1055	<0.0001	0.0008	0.2017	0.9967	0.5711	0.1087	<0.0001
StaffConfirmedCovid19	0.0306	0.0687	0.6558	0.0500	0.0286	0.0805	0.0271	0.0076	0.0004	0.0573	0.0409	0.1612	0.0337	0.0096	0.0004
Beds 50–100 vs. < 50	-	-	-	2.3038	0.8387	0.0060	1.1636	0.4415	0.0084	-	-	-	0.9696	0.3886	0.0126
Beds 100–249 vs. < 50	1.6802	0.5533	0.0526	2.6794	0.8157	0.0010	1.4548	0.4363	0.0009	0.4499	0.1721	0.0090	1.1814	0.3788	0.0018
Beds 250+ vs. < 50	1.2179	0.6283	0.0526	3.0326	0.8728	0.0005	1.6361	0.5335	0.0022	0.0351	0.4417	0.9367	0.9733	0.6406	0.1287
Veterans Homes	0.2716	1.4672	0.8531	3.2094	0.9753	0.0008	-	-	-	-	-	-	-	-	-
*SV-Socioeconomic	1.7252	0.9776	0.0776	-2.0068	0.5940	0.0007	0.0583	0.2620	0.8239	0.2679	0.4587	0.5592	0.1431	0.2961	0.6289
SV-Household characteristics	2.6002	0.6994	0.0002	1.3662	0.5024	0.0065	-0.0643	0.2316	0.7813	0.1171	0.4277	0.7837	-0.3994	0.2177	0.0666
SV-Race & Ethnicity	-0.2637	0.8883	0.7665	1.7283	0.5104	0.0007	0.0949	0.2147	0.6586	0.8216	0.3686	0.0258	0.3721	0.2581	0.5619
SV-Housing/transportation	-2.6812	0.7480	0.0003	-0.6008	0.5450	0.2703	-0.6201	0.2252	0.0059	1.0307	0.4032	0.0106	-0.1497	0.2581	0.5619
Urban Area	2.2458	0.5484	<0.0001	0.5436	0.2683	0.0428	-	-	-	-	-	-	-	-	-
Urban Cluster	-	-	-	-	-	-	-	-	-	-	-	-	-0.4387	0.1295	0.0007
Rural	-	-	-	-	-	-	-0.2799	0.1259	0.0262	0.4350	0.2323	0.0611	-	-	-
Inf_Intercept	2.1753	0.3509	<0.0001	2.2067	0.2166	<0.0001	2.1043	0.1141	<0.0001	2.5505	0.1592	<0.0001	2.4436	0.1211	<0.0001
Inf_AdmissionsCovid19	-0.5243	0.2037	0.0100	-0.1465	0.0759	0.0535	-1.0439	0.2281	<0.0001	-2.2767	0.5246	<0.0001	-2.1807	0.3821	<0.0001
Inf_SuspectedCovid19	0.2978	0.2138	0.1637	-0.1501	0.0886	0.0903	-0.2282	0.0740	0.0021	-0.0262	0.0531	0.6212	-0.6440	0.1758	0.0002
Inf_ConfirmedCovid19	-1.6278	0.5045	0.0013	-1.4553	0.4025	0.0003	-1.1916	0.1539	<0.0001	-2.7879	0.4993	<0.0001	-1.2695	0.1735	<0.0001
Alpha	2.8672	0.5006	<0.0001	3.2027	0.6827	<0.0001	2.6087	0.2174	<0.0001	6.5982	0.8795	<0.0001	2.1425	0.1952	<0.0001
Observed Mean	96.88			95.74			91.22			97.00			90.43		
Modelled Mean	96.89			95.82			91.17			97.05			90.57		
AIC	948			1700			5720			3072			4569		
SBC	1061			1826			5853			3201			4697		

^1^Intercept Negative Binomial Model to estimate COVID-19 death counts offset by facility’s occupancy; Inf_Intercept logistic models to estimate zeros (SAS v.9.3) controlling for testing and presence vs. absence of a ventilatory unit. ^2^SV = Social Vulnerability.

## Discussion

This study analyzed the CMS data on COVID-19 cases and deaths in nursing homes from 1-January to 26-July, 2020. Weekly maps were created to demonstrate regional COVID-19 spatial diffusion. Risk factors for COVID-19 death in nursing homes within four time periods revealed three universal findings.

The first universal risk factor for COVID-19 nursing home deaths were staff confirmed COVID-19 occurring across HHS Regions 1, 2, 3, 5 and 9 (1-January to 24-May, 2020), HHS Regions 1, 2, 3, 4 and 5 (25-July to 14-June, 2020), HHS Regions 3, 5 and 6 (15-June to 5-July, 2020) and HHS Regions 4 and 6 (6-July to 26-July, 2020). On May 29, 2020 “Considerations for Preventing the Spread of COVID-19 in Assisted Living Facilities” was published by the CDC [27]. Testing protocols during this time period were based on staff’s signs and symptoms consistent with COVID-19 (fever or chills, cough, shortness of breath, fatigue, muscle or body aches, headache, new loss of taste or smell, sore throat, congestion or runny nose, nausea or vomiting and/or diarrhea) [28]. Other guidelines for supply and the use of personal protective equipment (PPE) -e.g., eye protection, facemasks, gowns, and gloves were all in-place by 26-July, 2020. McGarry et al. (2020) [7] however, found that between 18-May and 19-July, 2020 there were severe shortages of PPE in addition to staff shortages, which may in part be explained by staff diagnosed with COVID-19. In previous literature nursing shortages were not observed in mid-April (April 22–29, 2020) in 23 states [14] findings consistent with this study from 1-January to 14-June, 2020. This study observed nursing shortages as early as 25-May, 2020 in HHS Region 5 and subsequently through 5-July, 2020 in HHS Regions 2, 4 and 5 likely due to the high infection rates among staff and/or patients at this time. These findings were consistent with Sugg et al. (2021) [8] who found significant shortages of staff working in nursing homes with COVID-19 patients through 30-June, 2020 with this study showing moderately significant shortages among Registered Nursing staff and highly significant shortages among aide staff, findings that are informative in further understanding the type of nursing staff shortages. In this study, nursing shortages were also observed from 6-July to 26-July, 2020 in HHS Regions 4 and 6 about three-weeks after the observed north-south diffusion. According to Hege et al (2022) [9] the nursing shortages continued to be a significant risk factor for COVID-19 infection rates beyond 26-July, 2020 of the pandemic using CMS COVID-19 data through 31-January, 2021.

A second universal finding was that the transfer of COVID-19 patients from hospitals into nursing homes had a significant impact on nursing home COVID-19 related deaths in HHS Regions 2 and 5 (1-January to 24-May, 2020) and HHS Region 5 (25-May to 14-June, 2020). From 1-January to 24-May, 2020) the case-fatality rates in HHS Region 2 were highest in New York (44.4) and HHS Region 5 in Michigan (36.4). From 25-May to 14-June, 2020 the case-fatality rates in HHS Region 2 were highest in New Jersey (31.0) and New York (22.3) and HHS Region 5 Michigan (63.6), Minnesota (46.8) and Illinois (38.4). Following the north-south diffusion of COVID-19 the practice of transferring hospitalized patients with COVID-19 into nursing homes for additional care and its effect on receiving nursing home deaths was significant in HHS Regions 4 and 6 (15-June to 5-July, 2020) and HHS Regions 3 and 4 (6-July to 26-July, 2020). The subsequent case fatality-rates in states in the South however, were substantially lower than in the Northeast. These noteworthy findings suggests that although the practice of transferring COVID-19 patients from hospitals into nursing homes continued over time in the South, other practices must have been in place to avoid high case-fatality rates.

Finally, nursing home location and type of neighborhood social vulnerability did not emerge as a significant risk factor for COVID-19 deaths until after the beginning of the pandemic, indicative of early undetected COVID-19 transmission across nursing homes country-wide. Thereafter, nursing homes in urban areas were the first to show high case-fatality rates for COVID-19 among residents. The two most common types of social vulnerability were neighborhoods with high-minority and non-English speaking residents and neighborhoods with a high proportion of dependents, single-family households and households with disabilities. These findings are similar to studies by Sugg et al. [8] who found important county-level characteristics significant for nursing home COVID-19 infection, including higher unemployment, higher average gross rent, higher percentage of occupied houses with no vehicle available and higher percent African American residents. Traverse et al. [15] also found county-level resources, rurality and counties with a high proportion of Black residents in part explained high rates of COVID-19 and deaths in nursing homes nationwide. Chatterjee et al. [14], Hege et al. [9] and Sugg et al. [8] found an increase in COVID-19 reporting in nursing homes in counties with high levels of COVID-19 infection, thus future research could focus on understanding the composition of populations in counties with high infection rates to assess minority population groups and households with disabilities to assess their increased vulnerability for COVID-19 transmission.

### Limitations

The limitations to this study include, first, the Nursing Home COVID-19 Public File includes data reported by all certified Medicare skilled nursing homes and Medicaid nursing facilities to the National Healthcare Safety Network Long Term Care Facility Module [18]. These data therefore do not include CMS assisted living facilities or intermediate care facilities for individuals with intellectual disabilities or private nursing homes that do not participate in CMS. Second, the nursing home surveillance data may include delayed reports and thus, the findings may change slightly as the module’s data are updated. This study utilized the original data for surveillance from 1-January to 26-July, 2020 provided by the CDC-CMS [4] to get a snapshot of the beginning of the COVID-19 epidemic in nursing homes in the U.S., the same data used to make early policy and programmatic healthcare decisions. Future research could reanalyze these data using current surveillance data on nursing homes to detect any potential differences in reporting and results. Third, those residents confirmed with COVID-19 who were transferred to hospitals, may have died in the hospital and therefore, were not counted in these data resulting in an underestimation of COVID-19 infection/transmission in nursing homes. This limitation may further explain the variation in COVID-19 cases and deaths across time-periods in the highly populated HHS Region 2. Fourth, the transmission of COVID-19 between nursing home residents and staff and vice versa and subsequent staff shortages are not known. Future research may be able to disentangle these questions using testing results in nursing homes with high-reporting data over time. Finally, future research could compare these results with state-specific nursing home COVID-19 data to learn more about the decentralization of COVID-19 healthcare policies and practices, in relation to federal guidelines, including facility-level quality assurance ratings.

## Conclusions

Studying COVID-19 in nursing homes from 1-January to 26-July, 2020 provided an opportunity to observe early regional trends in diffusion and risk factors for premature COVID-19 related deaths. This study found an early north-south diffusion of COVID-19 in nursing homes suggesting that improvements in communication between Health and Human Service administrators and the timely transmission of “Lessons Learned” to state health departments will further help to prevent premature nursing home deaths in future pandemics.

## Supporting information

S1 Table Nursing homes COVID-19 confirmed cases, deaths, and case-fatality rates^1^ by state^2^ and reporting weeks within Health and Human Service (HHS) Regions, 1-January to 26-July 2020. ^1^Case-fatality rates (deaths per 100 confirmed COVID-case residents). COVID-19 deaths < 20 or COVID-19 cases < 50 case-fatality rates not shown. ^2^Excludes Puerto Rico, Hawaii, Guam, and Alaska.
(DOCX)

## References

[1] Centers for Disease Control and Prevention (CDC). Guidance for limiting the transmission of COVID-19 for nursing homes. National Center for Health Statistics. 2020. Available from https://www.cdc.gov/coronavirus/2019-ncov/hcp/guidance-risk-assessment-hcp.html.

[2] Centers for Medicare & Medicaid. Guidance for infection control and prevention of Coronavirus Disease 2019 (COVID-19) in nursing homes (REVISED). 2020. Available from http://www.cms.gov/files/document/qso-20-14-nh-revised.pdf?eType=EmailBlastContent&eId=23036eb9-2439-42d1-a014-eebc7664544e.

[3] Centers for Disease Control and Prevention (CDC). Infection control guidance for healthcare professionals about Coronavirus (COVID-19). 2020. Available from https://www.cdc.gov/coronavirus/2019-ncov/hcp/infection-control.html.

[4] Centers for Medicare & Medicaid. Covid-19 Nursing home data. Department of Health & Human Services. 2020. Available from Data.CMS.gov: https://data.cms.gov/stories/s/bkwz-xpvg.

[5] Abrams HR, Loomer L, Gandhi A, Grabowski DC. Characteristics of U.S. nursing homes with Covid-19 cases. JAGS. 2020;68(8):1653–1656. doi: 10.1111/jgs.16661 32484912 PMC7300642

[6] White EM, Kosar CM, Feifer RA, Blackman C, Gravenstein S, Ouslander J, et al. Variation in SARS-CoV-2 prevalence in U.S. skilled nursing facilities. JAGS. 2020;68(10):2167–2173. doi: 10.1111/jgs.16752 32674223 PMC7404330

[7] McGarry BE, Grabowski DC, Barnett ML. Severe staffing and personal protective equipment shortage faced by nursing homes during the COVID-19 pandemic. Health Aff. (Millwood). 2020;39(10):1812–1821. 32816600 10.1377/hlthaff.2020.01269 PMC7598889

[8] Sugg MM, Spaulding TJ, Lane SJ, Runkle JD, Harden SR, Hege A, et al. Mapping community-level determinants of COVID-19 transmission in nursing homes: A multi-scale approach. Sci. Total Environ. 2021;752:141946 doi: 10.1016/j.scitotenv.2020.141946 32889290 PMC7446707

[9] Hege A, Lane S, Spaulding T, Sugg M, Iyer LS. County-level social determinants of health and COVID-19 in nursing homes, United States, June 1, 2020-January 31, 2021. Public Health Rep. 2022;37(1):137–148. doi: 10.1177/00333549211053666 34788163 PMC8721753

[10] Iyanda AE, Boakye KA. A 2-year pandemic period analysis of facility and county-level characteristics of nursing home coronavirus deaths in the United States, January 1, 2020-December 18, 2021. Geriatr. Nurs. 2022;44:237–244. doi: 10.1016/j.gerinurse.2022.02.013 35248837 PMC8858698

[11] Li Y, Temkin-Greener H, Shan G, Cai X. COVID-19 infections and deaths among Connecticut nursing home residents: facility correlates. JAGS. 2020;68(9):1899–1906. doi: 10.1111/jgs.16689 32557542 PMC7323378

[12] Harrington C., Chapman S., Spurlock B., Bakerjian D. Nurse staffing and coronavirus infections in California nursing homes. Policy Politics Nurs. Prac. 2020;21(3):174–186. doi: 10.1177/1527154420938707 32635838

[13] He M, Li Y, Fang F. Is there a link between nursing home reported quality and COVID-19 cases? Evidence from California skilled nursing facilities. JAMDA. 2020;21(7):905–908. doi: 10.1016/j.jamda.2020.06.016 32674817 PMC7294249

[14] Chatterjee P, Kelly S, Qi M, Werner RM. Characteristics and quality of U.S. nursing home reporting cases of Coronavirus Disease 2019 (COVID-19). JAMA Network Open. 2020;3(7):e2016930. doi: 10.1001/jamanetworkopen.2020.16930 32725243 PMC8310566

[15] Travers JL, Agarwal M, Estrada LV, Dick AW, Gracner T, Wu B, Stone PW. Assessment of Coronavirus Disease 2019 infection and mortality rates among nursing homes with different proportions of Black residents. JAMDA. 2021;22:893–898. doi: 10.1016/j.jamda.2021.02.014 33762185 PMC7898962

[16] McMichael TM, Currie DW, Clark S, Pogosjans S, Kay M, Schwartz NG, et al. Epidemiology of Covid-19 in a long-term care facility in King County, Washington. NEJM. 2020;382(21):2005–2011. doi: 10.1056/NEJMoa2005412 32220208 PMC7121761

[17] U.S. Department of Health and Human Services. HHS Regional Offices. 2021. Available from HHS.gov.

[18] Centers for Disease Control and Prevention (CDC). Nursing homes & long-term care facilities. Coronavirus Disease. 2020. Available from https://www.cdc.gov/coronavirus/2019-ncov/hcp/nursing-home-long-term-care.html.

[19] Flanagan BE, Gregory EW, Hallisey EJ, Heitgerd JL, Lewis B. A social vulnerability Index for disaster management. JHSEM. 2011;8(1). doi: 10.2202/1547-7355.1792

[20] Centers for Disease Control and Prevention (CDC). Social vulnerability index. 2020. Agency for Toxic Substances and Disease Registry. Data & Tools Download. Available from https://svi.cdc.gov/data-and-tools-download.html.

[21] U.S. Bureau of the Census, 2019 TIGER Boundary Files. Available from https://www.census.gov/geographies/mapping-files/time-series/geo/tiger-line-file.html.

[22] Environmental Systems Research Inc. (ESRI), 2022. Available from https://www.esri.com.

[23] American Society of Planning Officials. Nursing Homes, 1964. Available from https://www.planning.org/pas/reports/report185.htm.

[24] Hua HE, Tang W, Wang W, Crits-Christoph P. Structural zeros and zero-inflated models. Shanghai Arch. Psychiatry. 2014;26(4):236–242. 25317011 10.3969/j.issn.1002-0829.2014.04.008 PMC4194007

[25] SAS Institute. 2020. Available from https://www.sas.com/en_us/home.html.

[26] Erdman D, Jackson L, Sinko A. Zero-inflated Poisson and zero-inflated negative binomial models using he COUNTREG procedure. SAS Institute, Inc., Cary, NC.

[27] Centers for Disease Control and Prevention (CDC). Consideration for preventing spread of COVID-19 in assisted living facilities. 2020. Available from https://www.cdc.gov/coronavirus/2019-ncov/hcp/assisted-living.html.

[28] Centers for Disease Control and Prevention (CDC). Diagnostic testing. 2021. Available from https://www.cdc.gov/coronavirus/2019-ncov/hcp/nursing-homes-testing.html.

