# Supplementary material for: COVID-19 in nursing homes: Geographic diffusion and regional risk factors from January 1 to July 26, 2020 of the pandemic
Source: PLoS One. 2024 Aug 15;19(8):e0308339. doi: 10.1371/journal.pone.0308339 (PMC11326555; doi:10.1371/journal.pone.0308339)
Supplement: S1 Table — 1Case-fatality rates (deaths per 100 confirmed COVID-case residents). COVID-19 deaths < 20 or COVID-19 cases < 50 case-fatality rates not shown. 2Excludes Puerto Rico, Hawaii, Guam, and Alaska. (DOCX) [file pone.0308339.s001.docx]

Table 1S Nursing Homes COVID-19 Confirmed Cases, Deaths, and Case-Fatality Rates^1^ by State^2^ and Surveillance 24-May 24 to 26-July, 2020 within Health and Human Service (HHS) Regions.

|  | Week 1 | | Case | Weeks 2-3-4 | | Case | Weeks 5-6-7 | | Case | Weeks 8-9-10 | | Case | Deaths | Cases | Case |
| --- | --- | --- | --- | --- | --- | --- | --- | --- | --- | --- | --- | --- | --- | --- | --- |
| State | Deaths | Cases | Fatality | Deaths | Cases | Fatality | Deaths | Cases | Fatality | Deaths | Cases | Fatality | Total | Total | Fatality |
| **Region 1** | No. | No. | Rate^1^ | No. | No. | Rate^1^ | No. | No. | Rate^1^ | No. | No. | Rate^1^ | No. | No. | Rate^1^ |
| Connecticut | 1473 | 5254 | 28.0 | 266 | 761 | 34.95 | 58 | 177 | 32.77 | 20 | 81 | 24.69 | 1817 | 6273 | 28.97 |
| Massachusetts | 2598 | 8347 | 31.1 | 539 | 1035 | 52.08 | 279 | 270 | 103.33 | 94 | 117 | 80.34 | 3510 | 9769 | 35.93 |
| Maine | 21 | 123 | 17.1 | 9 | 9 | -- | 5 | 66 | -- | 10 | 22 | -- | 45 | 220 | 20.45 |
| New Hampshire | 116 | 470 | 24.7 | 79 | 308 | 25.65 | 40 | 159 | 25.16 | 14 | 14 | -- | 249 | 951 | 26.18 |
| Rhode Island | 353 | 1291 | 27.3 | 133 | 275 | 48.36 | 43 | 116 | 37.07 | 19 | 41 | -- | 548 | 1723 | 31.80 |
| Vermont | 31 | 98 | 31.6 | 0 | 0 | -- | 1 | 1 | -- | 0 | 1 | -- | 32 | 100 | 32.00 |
| Sub-Total | 4592 | 15583 | 29.5 | 1026 | 2388 | 42.96 | 426 | 789 | 53.99 | 157 | 276 | 56.88 | 6201 | 19036 | 32.58 |
| **Region 2** |  |  |  |  |  |  |  |  |  |  |  |  |  |  |  |
| New Jersey | 2756 | 9489 | 29.0 | 613 | 1976 | 31.02 | 211 | 432 | 48.84 | 278 | 169 | 164.50 | 3858 | 12066 | 31.97 |
| New York | 3582 | 8063 | 44.4 | 672 | 3011 | 22.32 | 163 | 880 | 18.52 | 59 | 398 | 14.82 | 4476 | 12352 | 36.24 |
| Sub-Total^2^ | 6338 | 17552 | 36.1 | 1285 | 4987 | 25.77 | 374 | 1312 | 28.51 | 337 | 567 | 59.44 | 8334 | 24418 | 34.13 |
| **Region 3** |  |  |  |  |  |  |  |  |  |  |  |  |  |  |  |
| District Columbia | 74 | 330 | 22.4 | 10 | 116 | -- | 11 | 3 | -- | 5 | 10 | -- | 100 | 459 | 21.79 |
| Delaware | 173 | 563 | 30.7 | 51 | 175 | 29.14 | 16 | 32 | -- | 7 | 24 | -- | 247 | 794 | 31.11 |
| Maryland | 632 | 2929 | 21.6 | 308 | 1115 | 27.62 | 160 | 282 | 56.74 | 55 | 404 | 13.61 | 1155 | 4730 | 24.42 |
| Pennsylvania | 2216 | 7696 | 28.8 | 671 | 1515 | 44.29 | 399 | 1152 | 34.64 | 332 | 1045 | 31.77 | 3618 | 11408 | 31.71 |
| Virginia | 326 | 1347 | 24.2 | 130 | 543 | 23.94 | 81 | 312 | 25.96 | 83 | 441 | 18.82 | 620 | 2643 | 23.46 |
| West Virginia | 40 | 228 | 17.5 | 1 | 8 | -- | 2 | 5 | -- | 2 | 25 | -- | 45 | 266 | 16.92 |
| Sub-Total | 3461 | 13093 | 26.4 | 1171 | 3472 | 33.73 | 669 | 1786 | 37.46 | 484 | 1949 | 24.83 | 5785 | 20300 | 28.50 |
| **Region 4** |  |  |  |  |  |  |  |  |  |  |  |  |  |  |  |
| Alabama | 231 | 1037 | 22.3 | 83 | 526 | 15.78 | 99 | 1001 | 9.89 | 149 | 1277 | 11.67 | 562 | 3841 | 14.63 |
| Florida | 425 | 1742 | 24.4 | 302 | 803 | 37.61 | 292 | 1608 | 18.16 | 786 | 4721 | 16.65 | 1805 | 8874 | 20.34 |
| Georgia | 423 | 2538 | 16.7 | 269 | 860 | 31.28 | 198 | 833 | 23.77 | 238 | 1506 | 15.80 | 1128 | 5737 | 19.66 |
| Kentucky | 189 | 825 | 22.9 | 68 | 364 | 18.68 | 82 | 428 | 19.16 | 131 | 370 | 35.41 | 470 | 1987 | 23.65 |
| Mississippi | 173 | 715 | 24.2 | 197 | 682 | 28.89 | 52 | 466 | 11.16 | 116 | 961 | 12.07 | 538 | 2824 | 19.05 |
| North Carolina | 247 | 1212 | 20.4 | 198 | 702 | 28.21 | 107 | 388 | 27.58 | 126 | 769 | 16.38 | 678 | 3071 | 22.08 |
| South Carolina | 150 | 855 | 17.5 | 93 | 375 | 24.80 | 104 | 512 | 20.31 | 191 | 1151 | 16.59 | 538 | 2893 | 18.60 |
| Tennessee | 71 | 277 | 25.6 | 25 | 194 | 12.89 | 45 | 239 | 18.83 | 48 | 435 | 11.03 | 189 | 1145 | 16.51 |
| Sub-Total | 1909 | 9201 | 20.8 | 1235 | 4506 | 27.41 | 979 | 5475 | 17.88 | 1785 | 11190 | 15.95 | 5908 | 30372 | 19.45 |
| **Region 5** |  |  |  |  |  |  |  |  |  |  |  |  |  |  |  |
| Illinois | 1451 | 6231 | 23.3 | 677 | 1764 | 38.38 | 311 | 919 | 33.84 | 159 | 428 | 37.15 | 2598 | 9342 | 27.81 |
| Indiana | 573 | 2031 | 28.2 | 246 | 710 | 34.65 | 243 | 357 | 68.07 | 334 | 354 | 94.35 | 1396 | 3452 | 40.44 |
| Michigan | 984 | 2706 | 36.4 | 418 | 657 | 63.62 | 141 | 189 | 74.60 | 38 | 311 | 12.22 | 1581 | 3863 | 40.93 |
| Minnesota | 273 | 1174 | 23.3 | 205 | 438 | 46.80 | 86 | 174 | 49.43 | 39 | 103 | 37.86 | 603 | 1889 | 31.92 |
| Ohio | 769 | 2627 | 29.3 | 315 | 1137 | 27.70 | 173 | 794 | 21.79 | 193 | 904 | 21.35 | 1450 | 5462 | 26.55 |
| Wisconsin | 77 | 557 | 13.8 | 66 | 219 | 30.14 | 29 | 76 | 38.16 | 22 | 90 | 24.44 | 194 | 942 | 20.59 |
| Sub-Total | 4127 | 15326 | 26.9 | 1927 | 4925 | 39.13 | 983 | 2509 | 39.18 | 785 | 2190 | 35.84 | 7822 | 24950 | 31.35 |
| **Region 6** |  |  |  |  |  |  |  |  |  |  |  |  |  |  |  |
| Arkansas | 39 | 335 | 11.6 | 26 | 199 | 13.07 | 70 | 460 | 15.22 | 62 | 250 | 24.80 | 197 | 1244 | 26.55 |
| Louisiana | 597 | 2411 | 24.8 | 263 | 934 | 28.16 | 198 | 638 | 31.03 | 315 | 1378 | 22.86 | 1373 | 5361 | 20.59 |
| New Mexico | 52 | 225 | 23.1 | 14 | 63 | -- | 6 | 62 | -- | 52 | 129 | 40.31 | 124 | 479 | 35.68 |
| Oklahoma | 78 | 375 | 20.8 | 23 | 134 | 17.16 | 19 | 108 | -- | 35 | 223 | 15.70 | 155 | 840 | 21.35 |
| Texas | 341 | 2002 | 17.0 | 200 | 1163 | 17.20 | 333 | 2234 | 14.91 | 896 | 5778 | 15.51 | 1770 | 11177 | 18.62 |
| Sub-Total | 1107 | 5348 | 20.7 | 526 | 2493 | 21.10 | 626 | 3502 | 17.88 | 1360 | 7758 | 17.53 | 6562 | 28522 | 23.01 |
| **Region 7** |  |  |  |  |  |  |  |  |  |  |  |  |  |  |  |
| Iowa | 180 | 770 | 23.4 | 104 | 223 | 46.64 | 41 | 176 | 23.30 | 90 | 307 | 29.32 | 415 | 1476 | 28.12 |
| Kansas | 46 | 162 | 28.4 | 25 | 75 | 33.33 | 24 | 129 | 18.60 | 33 | 116 | 28.45 | 128 | 482 | 26.56 |
| Missouri | 247 | 1014 | 24.4 | 119 | 441 | 26.98 | 78 | 375 | 20.80 | 80 | 623 | 12.84 | 524 | 2453 | 21.36 |
| Nebraska | 68 | 285 | 23.9 | 39 | 69 | 56.52 | 27 | 41 | 65.85 | 22 | 62 | 35.48 | 156 | 457 | 34.14 |
| Sub-Total | 541 | 2231 | 24.3 | 287 | 808 | 35.52 | 170 | 721 | 23.58 | 225 | 1108 | 20.31 | 1223 | 4868 | 25.12 |
| **Region 8** |  |  |  |  |  |  |  |  |  |  |  |  |  |  |  |
| Colorado | 299 | 863 | 34.7 | 74 | 281 | 26.33 | 49 | 117 | 41.88 | 22 | 81 | 27.16 | 444 | 1342 | 33.08 |
| Montana | 2 | 5 | -- | 0 | 0 | -- | 1 | 1 | -- | 0 | 4 | -- | 3 | 10 | -- |
| North Dakota | 28 | 109 | 25.7 | 10 | 18 | -- | 1 | 9 | -- | 4 | 28 | -- | 43 | 164 | 26.22 |
| South Dakota | 21 | 17 | -- | 5 | 31 | -- | 7 | 28 | -- | 7 | 43 | -- | 40 | 119 | 33.61 |
| Utah | 9 | 42 | -- | 10 | 73 | -- | 15 | 169 | -- | 45 | 297 | 15.15 | 79 | 581 | 13.60 |
| Wyoming | 2 | 10 | -- | 1 | 6 | -- | 0 | 1 | -- | 0 | 0 | -- | 3 | 17 | -- |
| Sub-Total | 361 | 1046 | 34.5 | 100 | 409 | 24.45 | 73 | 325 | 22.46 | 78 | 453 | 17.22 | 612 | 2233 | 27.41 |
| **Region 9** |  |  |  |  |  |  |  |  |  |  |  |  |  |  |  |
| Arizona | 94 | 287 | 32.8 | 83 | 442 | 18.78 | 113 | 737 | 15.33 | 139 | 668 | 20.81 | 429 | 2134 | 20.10 |
| California | 1172 | 5212 | 22.5 | 714 | 2592 | 27.55 | 462 | 2932 | 15.76 | 453 | 3435 | 13.19 | 2801 | 14171 | 19.77 |
| Nevada | 86 | 453 | 19.0 | 11 | 88 | -- | 6 | 93 | -- | 15 | 139 | -- | 118 | 773 | 15.27 |
| Sub-Total^3^ | 1352 | 5952 | 22.7 | 808 | 3122 | 25.88 | 581 | 3762 | 15.44 | 607 | 4242 | 14.31 | 3348 | 17078 | 19.60 |
| **Region 10** |  |  |  |  |  |  |  |  |  |  |  |  |  |  |  |
| Idaho | 27 | 55 | 49.1 | 10 | 2 | -- | 2 | 33 | -- | 11 | 148 | -- | 50 | 238 | 21.01 |
| Oregon | 19 | 98 | 19.4 | 0 | 14 | -- | 1 | 25 | -- | 4 | 70 | -- | 24 | 207 | 11.59 |
| Washington | 195 | 665 | 29.3 | 39 | 125 | 31.20 | 30 | 145 | 20.69 | 40 | 295 | 13.56 | 304 | 1230 | 24.72 |
| Sub-Total^4^ | 241 | 818 | 29.5 | 49 | 141 | 34.75 | 33 | 203 | 16.26 | 55 | 513 | 10.72 | 378 | 1675 | 22.57 |
| National | 26972 | 95571 | 28.2 | 8414 | 27251 | 30.88 | 4914 | 20384 | 24.11 | 5873 | 30246 | 19.42 | 46173 | 173452 | 26.62 |

^1^Case-fatality rates (deaths per 100 confirmed COVID-case residents). COVID-19 deaths < 20 or COVID-19 cases < 50 case-fatality rates not shown.

^2^Excludes Puerto Rico, Hawaii, Guam, and Alaska.
